# Use of Lipid-Containing Nanoemulsion Artificial Tears in Patients With Combined Dry Eye Disease and Meibomian Gland Dysfunction: A Retrospective Cohort Study

**DOI:** 10.7759/cureus.90714

**Published:** 2025-08-22

**Authors:** Chia-Yi Lee, Chao Kai Chang, Yu-Ling Chang, Shun-Fa Yang, Chin-Te Huang

**Affiliations:** 1 Institute of Medicine, Chung Shan Medical University, Taichung, TWN; 2 Ophthalmology, Nobel Eye Institute, Taipei, TWN; 3 Pediatric Medicine, Cathay General Hospital, Taipei, TWN; 4 Medical Research, Chung Shan Medical University, Taichung, TWN

**Keywords:** artificial tear, dry eye disease, meibomian gland dysfunction, ocular surface stain, tear break-up time

## Abstract

Purpose: This study aimed to evaluate the effects of lipid-containing nanoemulsion artificial tears (ATs) on tear film stability, ocular surface health, and symptoms in patients with dry eye disease (DED) and meibomian gland dysfunction (MGD).

Methods: A retrospective cohort study was conducted on patients who received either lipid-containing nanoemulsion ATs (study group, 50 eyes) or another AT formulation (control group, 50 eyes). Primary outcomes were non-invasive tear break-up time (NITBUT), Schirmer I test, ocular surface staining, meibomian gland loss rate, and DED-related symptoms. Data were analyzed using independent t-tests and generalized linear models.

Results: NITBUT was significantly longer in the study group compared with the control group (P < 0.001). Ocular surface staining and DED-related symptoms were also significantly better in the study group (both P < 0.05). The magnitude of improvement in NITBUT, meibomian gland loss rate, ocular surface staining, and DED-related symptoms was greater in the study group than in the control group (all P < 0.05). Sensitivity analysis showed that male sex was associated with DED improvement in the study group (P = 0.038), while both younger age and male sex were associated with improvement in the control group (both P < 0.05).

Conclusion: Lipid-containing nanoemulsion ATs were associated with greater improvements in tear film stability and ocular surface outcomes in patients with DED and MGD.

## Introduction

Dry eye disease (DED) is a common external eye disorder with subtypes that include aqueous deficiency, mucin deficiency, evaporative excess, and mixed forms [1]. The prevalence of DED in the Asia-Pacific region has been reported to exceed 15% [2]. Typical symptoms include dryness, fatigue, foreign body sensation, burning, photophobia, grittiness, and redness [3]. Severe or refractory DED may impair visual acuity and quality of life [4] and can lead to superficial keratitis [5].

Management options for DED range from medical to surgical approaches. Artificial tears (ATs) remain the mainstay of treatment [6,7], while other eyedrops such as topical steroids have shown additional benefit with acceptable safety [8]. In cases unresponsive to medical therapy, surgical interventions such as amniotic membrane transplantation may be required [9]. Over the past decade, hyaluronic acid has been widely incorporated into AT formulations due to its efficacy in advanced DED [10].

Lipid-containing nanoemulsion ATs have recently been introduced and shown promising efficacy with a low rate of adverse effects [11]. These formulations appear effective across various subtypes of DED, including evaporative, aqueous-deficient, and mixed types [12,13]. However, their role in patients with ocular comorbidities remains unclear. Meibomian gland dysfunction (MGD), a frequent contributor to DED, can exacerbate symptoms and disease severity [14], highlighting the need to assess the efficacy of lipid-containing nanoemulsion ATs in this subgroup.

This retrospective cohort study aimed to (1) compare the effects of lipid-containing nanoemulsion ATs and conventional ATs on tear film stability, ocular surface health, and symptoms in patients with concurrent DED and MGD, and (2) identify demographic predictors of treatment response.

## Materials and methods

Ethics declaration

This study adhered to the principles of the Declaration of Helsinki (1964) and its subsequent amendments. Approval was obtained from the Research Ethics Committee of the National Changhua University of Education (project code: NCUEREC-113-056; approval date: 07/03/2024). The requirement for written informed consent was waived by the Committee due to the retrospective nature of the study.

Participant selection

This retrospective cohort study was conducted at the Nobel Eye Institute, an ophthalmic institution operating more than 20 clinics across Taiwan. The study period was from December 10, 2024, to April 15, 2025. Eligible participants met all of the following criteria: (1) age 20-100 years, (2) diagnosed with DED at any Nobel Eye Institute clinic, (3) underwent automatic ocular surface analyzer testing at a clinic visit, (4) received DED-related therapy exclusively at Nobel Eye Institute clinics, as confirmed through the electronic medical records of the Taiwan Health Insurance Administration, and (5) attended follow-up visits for at least three months. Exclusion criteria included a history of (1) central corneal erosion, (2) corneal ulcer, (3) central corneal opacity, (4) deep corneal infiltration, (5) chemical injury involving the central cornea, (6) corneal perforation, (7) keratorefractive surgery, (8) cicatricial conjunctivitis, (9) systemic inflammatory disease (e.g., Sjögren’s syndrome, rheumatoid arthritis, diabetes mellitus, systemic lupus erythematosus, thyroid eye disease), or (10) use of more than one artificial tear (AT) formulation during the follow-up period. Participants were assigned to groups according to the prescribed AT formulation. For consistency, only the right eye of each participant was analyzed, following our previous methodology [10]. After selection, 50 eyes were included in the study group (lipid-containing nanoemulsion ATs) and 50 in the control group (another AT formulation).

Artificial tear usage

The study group received a lipid-containing nanoemulsion AT (Systane® Complete, Alcon, Fort Worth, TX, USA), while the control group received another AT formulation (Systane® Balance, Alcon, Fort Worth, TX, USA). Participants in both groups were instructed to instill the assigned ATs 2-4 times daily without a drug holiday. If dryness or other DED-related symptoms persisted, use could be increased to a maximum of 10 times daily due to the preservatives in both ATs. In addition, all participants received steroid eyedrops and an overnight ointment as adjuvant treatment. The steroid eyedrop was 0.02% fluorometholone (Foxone 0.02%, Winston Medical Supply Co., Ltd., Tainan City, Taiwan), prescribed four times daily. The overnight ointment was 0.2% carbomer gel (Ginpol Gel 0.2%, Winston Medical Supply Co., Ltd., Tainan City, Taiwan), applied before sleep. Additional use of these two medications outside the prescribed regimen was not recommended.

Dry eye evaluation

All participants underwent routine DED-related examinations before and after treatment, using a standardized measurement protocol across all clinics. Any prior ophthalmic diseases or surgeries were recorded. Pre-treatment examinations included the following: (i) uncorrected distance visual acuity (UDVA), measured using a Snellen chart; (ii) non-invasive tear break-up time (NITBUT) and meibomian gland loss rate, assessed with an automatic ocular surface analyzer (SBM Device, SBM Sistemi, Strada Torino, Orbassano, Italy). For this procedure, patients sat in front of the analyzer, which recorded a 20-second video of the ocular surface. Patients were instructed not to blink during filming. The software then automatically calculated NITBUT and meibomian gland loss. Meibomian gland loss was graded using the meiboscore system: Grade 0 (no loss), Grade 1 (less than one-third of the area lost), Grade 2 (one-third to two-thirds lost), and Grade 3 (more than two-thirds lost); (iii) Schirmer I test, patients rested for 10 seconds after topical anesthesia. A Schirmer strip was placed at the lateral one-third of the lower eyelid, and patients kept their eyes closed for five minutes. The strip was then removed and the length of wetting measured; (iv) fluorescein ocular surface staining, a fluorescein strip was swept across the inferior bulbar conjunctiva. After patients closed their eyes for ~5 seconds, staining of the cornea and conjunctiva was evaluated under a slit-lamp biomicroscope with a blue filter. Severity was graded using the Oxford Scheme [15]; and (v) DED-related symptoms, including dryness, burning, itch, soreness, grittiness, fatigue, photophobia, redness, foreign body sensation, and discharge, which were extracted from medical records. Post-treatment examinations included UDVA, fluorescein staining, NITBUT, meibomian gland loss rate, and the Schirmer I test. Persistent DED-related symptoms after AT therapy were also recorded. DED-related examinations conducted prior to treatment and up to three months after therapy were analyzed. There were no missing data.

Statistical analysis

Statistical analyses were performed using IBM SPSS Statistics for Windows, Version 20.0 (Released 2011; IBM Corp., Armonk, NY, United States). The statistical power of this study was 0.87, with an alpha of 0.05 and a medium effect size, calculated using G*Power version 3.1.9.2 (Heinrich Heine Universität, Düsseldorf, Germany). The Shapiro-Wilk test was used to assess data normality, and the results confirmed that the data were normally distributed (all P > 0.05). Descriptive statistics were applied to summarize baseline characteristics. Pre-treatment and post-treatment comparisons between the study and control groups were conducted using the independent t-test and chi-square test. To evaluate the effects of different AT treatments on DED improvement, a generalized linear model was applied, analyzing changes in NITBUT, DED-related symptoms, meibomian gland loss rate, ocular surface staining, and Schirmer I test results, while adjusting for age, sex, steroid eyedrop use, and overnight ointment use. Adjusted odds ratios (aORs) with corresponding 95% confidence intervals (CIs) were derived from multivariable analysis. In subgroup analyses, participants were stratified by younger age (<40 years) and male sex, and the generalized linear model was applied again to examine the influence of these factors on treatment outcomes. DED improvement was defined as meeting any of the following: (1) elimination of at least one DED-related symptom, (2) increase in NITBUT ≥ 2 seconds, (3) increase in Schirmer I test ≥ 1 mm, (4) absence of ocular surface staining, or (5) improvement in meibomian gland loss rate from grade 2-3 to grade 0-1. A value of P < 0.05 was considered statistically significant, and results with P < 0.001 were reported as P < 0.001.

## Results

The baseline characteristics of the study and control groups are summarized in Table 1. The mean age was 41.65 ± 7.42 years in the study group and 40.33 ± 6.86 years in the control group, with no significant difference (P = 0.357). The distributions of sex and pre-existing ocular disease were also comparable between groups (both P > 0.05). Regarding ophthalmic parameters, UDVA did not differ significantly between the groups (P = 0.367). Similarly, pre-treatment NITBUT, Schirmer I test results, meibomian gland loss rate, ocular surface staining, and DED-related symptoms showed no significant differences (all P > 0.05).

**Table 1:** Initial characteristics of the two groups. DED: dry eye disease, IOP: intraocular pressure, N: number, SD: standard deviation, NITBUT: non-invasive tear break-up time, UDVA: uncorrected distance visual acuity. Independent t-test and a chi-square test were used for the statistical analysis.

Feature	Study group (N = 50)	Control group (N = 50)	P-value	Statistic test
Age (years, mean ± SD)	41.65 ± 7.42	40.33 ± 6.86	0.357	Independent t-test
Sex (M:)	23:27	28:22	0.424	Chi-square test
Ocular disease	-	-	0.504	Chi-square test
Retinal degeneration	2	1	-	-
Glaucoma	0	2	-	-
Cataract	1	1	-	-
UDVA (LogMAR)	0.26 ± 0.12	0.24 ± 0.10	0.367	Independent t-test
Schirmer I test (mm)	8.46 ± 1.34	8.71 ± 1.28	0.343	Independent t-test
NITBUT (seconds)	6.31 ± 1.17	6.15 ± 1.08	0.479	Independent t-test
Meibomian gland loss rate	-	-	0.825	chi-square test
Grade 0-1	31	33	-	-
Grade 2-3	19	17	-	-
Ocular surface staining	-	-	0.833	Chi-square test
Absent	34	32	-	-
Present	16	18	-	-
DED-related symptoms	-	-	0.851	Chi-square test
0	0	0	-	-
1	27	25	-	-
≥2	23	25	-	-

After the three-month treatment period, UDVA remained comparable between the study and control groups (P = 0.068). NITBUT was significantly higher in the study group than in the control group (8.77 ± 1.20 vs. 7.23 ± 1.11, P < 0.001) (Table 2). The distributions of ocular surface staining and DED-related symptoms were also significantly better in the study group (P < 0.05 for both). In contrast, Schirmer I test results and meibomian gland loss rates did not differ significantly between the groups (both P > 0.05) (Table 2). With respect to the amplitude of improvement, the study group demonstrated significantly greater improvements in NITBUT, meibomian gland loss rate, ocular surface staining, and DED-related symptoms compared with the control group (all P < 0.05) (Table 3). The change in Schirmer I test results, however, remained similar between groups (P = 0.318) (Table 3).

**Table 2:** Treatment outcomes of the two groups after artificial tear therapy. DED: dry eye disease, SE: spherical equivalent, UDVA: uncorrected distance visual acuity. Independent t-test and a chi-square test were used for the statistical analysis. Asterisk (*) denotes a significant difference between groups.

Outcome	Study group (N = 50)	Control group (N = 50)	P-value	Statistic test
UDVA	0.23 ± 0.08	0.20 ± 0.08	0.068	Independent t-test
Schirmer I test	9.85 ± 1.31	10.04 ± 1.25	0.459	Independent t-test
NITBUT	8.77 ± 1.20	7.23 ± 1.11	<0.001*	Independent t-test
Meibomian gland loss rate	-	-	0.241	Chi-square test
Grade 0-1	41	35	-	-
Grade 2-3	9	15	-	-
Ocular surface staining	-	-	0.020*	Chi-square test
Absent	45	36	-	-
Present	5	14	-	-
DED-related symptoms	-	-	0.047*	Chi-square test
0	10	3	-	-
1	29	39	-	-
≥2	11	8	-	-

**Table 3:** Differences in dry eye improvement in the study group compared to the control group. aOR: adjusted odds ratio, CI: confidence interval, DED: dry eye disease, NITBUT: non-invasive tear break-up time. A generalized linear model was used for the statistical analysis. Asterisk (*) denotes significant improvement in the study group compared to the control group.

Outcome (reference: control group)	aOR	95% CI		P-value
		Lower	Upper	
Schirmer I test	1.06	0.94	1.19	0.318
NITBUT	1.23	1.02	1.44	0.005*
Meibomian gland loss rate	1.12	1.01	1.24	0.028*
Ocular surface staining	1.17	1.05	1.30	<0.001*
DED-related symptoms	1.25	1.07	1.43	<0.001*

In the sensitivity analysis, male sex was significantly associated with DED improvement in the study group (P = 0.038). In the control group, both younger age and male sex were associated with DED improvement (both P < 0.05) (Table 4).

**Table 4:** Demographic factors that correlated with better dry eye improvement in the study and control groups. aOR: adjusted odds ratio, CI: confidence interval. A generalized linear model was used for the statistical analysis. Asterisk (*) denotes significant correlation to dry eye improvement.

Factor	aOR	95% CI		P-value
		Lower	Upper	
Study group	-	-	-	-
Young age	1.04	0.96	1.12	0.594
Male sex	1.08	1.01	1.15	0.038*
Control group	-	-	-	-
Young age	1.10	1.03	1.18	0.009*
Male sex	1.17	1.05	1.29	<0.001*

## Discussion

In this study, patients using lipid-containing nanoemulsion ATs showed better post-treatment outcomes in NITBUT, ocular surface staining, and DED-related symptoms compared with those using another AT formulation. The magnitudes of improvement in NITBUT, ocular surface staining, meibomian gland loss rate, and DED-related symptoms were also greater in the lipid-containing nanoemulsion AT group. Additionally, male sex was associated with DED improvement in both groups, while younger age was associated with improvement only in the control group.

The development of DED involves multiple pathways [10,16]. Goblet cell damage from autoimmune disease or chemical exposure can lead to reduced mucin secretion and mucin-deficient DED, which is characterized by shortened tear film stability, corneal epithelial disruption, and bothersome symptoms [5,10,16]. Inflammatory cytokines also play a role, as elevated interleukin levels have been reported in patients with DED [17]. Impaired ocular surface homeostasis and reduced tear film stability further contribute to disease progression [1]. A decline in tear film stability causes epithelial damage and triggers inflammatory cytokine release, which raises tear film osmolarity and perpetuates a cycle of instability, commonly referred to as the vicious cycle of DED [18]. Oxidative stress is another important factor; excessive oxidative stress can damage DNA, induce lipid peroxidation, and promote DED episodes [19,20]. Lipid-containing ATs have demonstrated anti-inflammatory effects, which is clinically relevant given that inflammation is a major mechanism in DED development [21,22]. Moreover, the lipid component in nanoemulsion formulations can enhance tear film stability [23]. Considering these advantages, lipid-containing nanoemulsion ATs may be more effective than other AT formulations, particularly in patients with comorbidities such as MGD. The findings of the present study support this concept.

The use of lipid-containing nanoemulsion ATs was associated with greater DED improvement in participants with concurrent DED and MGD. A previous study on DED with comorbidities reported the efficacy of lipid-containing nanoemulsion ATs in reducing contact lens discomfort and DED-related symptoms [24]. However, few studies have specifically evaluated their effectiveness in patients with both DED and MGD. To our knowledge, the present study is among the first to demonstrate superior outcomes with lipid-containing nanoemulsion ATs in this population. The baseline characteristics of the study and control groups showed no significant differences, suggesting adequate group homogeneity. In addition, risk factors for DED, such as age and sex [25], were adjusted for in the generalized linear model, minimizing their confounding influence. Taken together, these findings suggest that lipid-containing nanoemulsion ATs may offer a more effective treatment option for DED compared to other AT formulations. MGD patients typically present with lipid dysregulation and ocular surface inflammation, both of which contribute to irritation symptoms [26]. The nanoemulsion technology may enhance lipid penetration and retention within the pre-corneal tear film [27], thereby addressing lipid deficiency and alleviating related symptoms. In our study, the amplitudes of DED improvement were significantly greater in the study group for most parameters, with the exception of the Schirmer I test. This finding aligns with the mechanism of action of lipid-containing nanoemulsion ATs, which primarily replenish the lipid layer and stabilize the tear film rather than directly influencing tear secretion [27]. The observed improvements in NITBUT and meibomian gland loss rate may represent moderate clinical significance, whereas the reduction of ocular surface staining in nearly 70% of eyes could indicate a more meaningful therapeutic effect. The rapid improvement in meibomian gland condition, however, warrants further investigation to clarify its underlying mechanism.

In the sensitivity analysis, male sex was associated with greater DED improvement in participants treated with lipid-containing nanoemulsion ATs. Female sex is a well-known predisposing factor for the development and progression of DED [25], and previous reports indicate that DED prevalence is approximately 1.5 times higher in women than in men [28]. This sex-related difference may explain the superior improvement observed in male participants receiving lipid-containing nanoemulsion ATs. In the control group using other AT formulations, both younger age and male sex were associated with better DED improvement. This finding differs slightly from the lipid-containing nanoemulsion AT group, and, to our knowledge, no prior studies have reported similar results. Age is another important risk factor for DED, with studies showing that men over 80 years of age have a significantly higher risk compared with those aged 50-54 years [29]. Interestingly, in our study, younger age was significantly associated with better outcomes in the control group but not in the lipid-containing nanoemulsion AT group. One possible explanation is that the stronger therapeutic effect of lipid-containing nanoemulsion ATs may have mitigated the influence of certain known risk factors, such as age, on treatment response. Furthermore, since the mean age of our study population was just above 40 years, the cohort did not include a sufficient number of “elderly” participants to demonstrate the expected age-related challenges in DED management.

DED is a common ocular disease worldwide, affecting about 3% of middle-aged men in the United States [29]. In East Asian populations, its prevalence may exceed 20% [25]. MGD is also frequent, reported in approximately 21% of individuals in the United States [30]. Since MGD is strongly associated with DED, patients with both conditions often experience greater disease severity than the general population [14]. Given their high prevalence and synergistic effects, identifying effective treatment strategies for DED in patients with concurrent MGD is particularly important.

This study has several limitations. First, the relatively small study population of 100 eyes may have introduced statistical bias, although the statistical power was acceptable. Second, the frequency of AT use was not strictly controlled. While no patient used ATs more than five times daily, variations in dosing and lack of adherence monitoring could have influenced outcomes and reduced reproducibility. Third, the retrospective design may have affected population homogeneity and accuracy, despite similar baseline characteristics between groups. Because of this design, standardized questionnaires were not applied to assess DED symptoms, limiting the precision and reliability of symptom evaluation. Additional factors, including inter-clinic measurement differences, potential sampling bias, one-eye analysis, uncontrolled confounders, short follow-up, and absence of blinding, may also have impacted the results. Finally, as all participants were Han Taiwanese, the generalizability of our findings to other populations is limited.

## Conclusions

The use of lipid-containing nanoemulsion ATs was associated with greater tear film stability and symptom relief in patients with concurrent DED and MGD. In addition, known risk factors for DED appeared to have less influence on treatment outcomes in this group. These findings suggest that lipid-containing nanoemulsion ATs may be a preferable option for DED patients with coexisting MGD. Larger prospective studies are warranted to confirm these results and to evaluate their effectiveness in patients with autoimmune disease or a history of keratorefractive surgery.
